# IL-23/IL-27 Ratio in Peripheral Blood of Patients with Breast Cancer

**Published:** 2014-07

**Authors:** Ali Khodadadi, Mahboobeh Razmkhah, Ali-Reza Eskandari, Ahmad Hosseini, Mojtaba Habibagahi, Abbas Ghaderi, Mansooreh Jaberipour

**Affiliations:** 1 Department of Immunology, Ahvaz Jondishapur University of Medical Sciences, Ahvaz, Iran;; 2 Institute for Cancer Research, School of Medicine, Shiraz University of Medical Sciences, Shiraz, Iran;; 3 Department of Immunology, School of Medicine, Shiraz University of Medical Sciences, Shiraz, Iran

**Keywords:** Neoplasms, Breast Neoplasm, Interleukin-23, Interleukin-27

## Abstract

**Background:** Interleukin (IL)-23 and IL-27 are two IL-12-related cytokines which their function may dramatically influence the inflammatory response to tumor development. IL-12 and IL-27 seem to have antagonistic roles with IL-23 in tumor site. In this study, IL-23 and IL-27 mRNA expressions were analyzed in peripheral blood of patients with breast cancer and healthy volunteers using quantitative real-time PCR.

**Methods: **Peripheral blood samples were collected from 50 women with breast cancer and 50 healthy ones. The total RNA was extracted from peripheral blood after lysis with ammonium chloride and TRizol reagent and the cDNA was synthesized. The expression of IL-23 and IL-27 gene transcripts was determined with real-time polymerase chain reaction (qRT-PCR) using Syber Green PCR Master Mix.

**Results:** It is found that IL-23 and IL-27 transcripts had significantly higher expression in peripheral blood of patients compared with the healthy controls. The ratio of IL-23 transcript expression to IL-27 was 3.4 fold lower in the studied patients compared with the normal individuals.

**Conclusion: **It is concluded that the over expression of IL-23 and IL-27 gene transcript in peripheral blood of breast cancer patients may be an immune response against tumor development and the inflammatory response plays a critical role in tumor development via up regulating the corresponding cytokines. However, the IL-23/IL-27 ratio may play an important role in cytokine-based immunotherapy against cancer. Further research should be carried out to assess these cytokines in a larger sample size. .

## Introduction

Cytokines administer a variety of biological effects with an important regulatory role in cell growth, survival and differentiation. It also indicates their contribution to the progression of malignancy. It is known that the formation of tumor cells relies on the oncogenic changes as well as the interaction between tumor cells and stromal environment. 

Understanding the role of cytokines in breast cancer has been the focal point of attention in the recent decades. Some cytokines stimulate breast cancer proliferation and some others modulate anti-tumor response. More recently, different cytokines have been identified with promising characteristics for future cancer immunotherapy.^1^ The members of Interleukin (IL)-12 family consisting of heterodimeric cytokines including IL-12 and IL-27, are the most potent antitumor cytokines.^2^^-^^4^ IL-27 consists of EBI3 (an IL-12p40 related protein) and p28 (an IL-12p35 related subunit). This cytokine activates both signal transducers and activator of transcription STAT1 and STAT3 through distinct IL-27 receptor subunits and enhances proliferation of naive CD4+T cells.^5^^,^^6^ It has been described that IL-27 is mostly produced by activated antigen presenting cells (APCs) and leads to priming and proliferation of naive CD4 T cells. Similar to IL-12, IL-27 is also able to prompt IFN-gamma production from naive CD4+ T cells^5^ IL-27 promotes the T helper cell (Th1) differentiation through up-regulating *T-b*ox *e*xpressed in *T* cells (T-bet)^7^ and Intercellular Adhesion Molecule 1 (ICAM-1).^6^ However, it inhibits the differentiation toward Th2,^8^^,^^9^ Th17 type responses^10^^,^^11^ and the production of pro-inflammatory cytokines.^12^^,^^13^ IL-27 together with the transforming growth factor beta (TGF-β) plays a critical role in generating IL-10-producing anti-inflammatory Type 1 regulatory T (Tr1) cells.^14^ IL-23 is a heterodimeric cytokine consisting of two subunits including p40 (also a subunit of the IL-12 cytokine) and p19 (a component of IL-23 alpha).^14^ IL-23 activates STAT3, acts on memory CD4+T cells, induces their proliferation and the production of cytokines such as IL-17 and IL-22.^15^^,^^16^ As a physiological function, IL-23 contributes to the development of the inflammatory Th17 cells^17^ and together with TGF-β and IL-6, to the induction of the Th17 differentiation.^18^^,^^19^ However, in the past the antagonistic roles of IL-12 and IL-27 with IL-23 in tumor have received considerable attention.^20^

Inflammatory responses play critical roles at different stages of tumor development as well as affecting immune surveillance and responses to therapy. As mentioned above, IL-23 induces the inflammatory responses through Th17. IL-23 production leads to the infiltration of neutrophils and macrophages and consequently to the chronic inflammation; an important process for tumor progression. In contrast, IL-12 and IL-27 act as inhibitors of Th2 type responses and play key roles in anti-tumor immune responses.^20^

Since the roles of inflammation are accepted in tumorigenesis, it is obvious that an inflammatory microenvironment is a necessary element of all tumors. Based on these reports, still little is known about specific IL-23 and IL-27 alterations associated with the breast cancer. Currently available data on this topic is controversial. Consequently, the assessment of changes in IL-23 and IL-27 expression in peripheral blood might provide supplementary information on the role of these cytokines in patients with breast cancer. It is purposed to investigate the mRNA expression of both IL-23 and IL-27 in the peripheral blood of patients diagnosed with breast cancer in relation with healthy controls and its association with clinico-pathological variables. Such results may provide a better insight on the interactions between tumor cells and the cells of the immune system. . 

## Patients and Methods

*Subjects*

The participants comprised 50 women with diagnosed infiltrating ductal carcinoma of the breast as confirmed by histopathological studies. Clinical stages of the disease were defined based on TNM staging as proposed by AJCC.^21^ The patients were referred to the laboratory of the breast clinic (Shiraz University of Medical Sciences, Shiraz, Iran) during 2011. Peripheral blood (2ml) with EDTA was collected before any clinical intervention such as surgery, radiotherapy, chemotherapy or immunotherapy. Patients with infectious or inflammatory diseases were excluded. Blood samples from 50 healthy female volunteers were obtained as a control group. Control subjects with a history of malignancies or autoimmune disorders were excluded from this study. It is worth mentioning that neither the patient nor the control individuals consumed any form of medication during the course of this study. Individuals in both groups were sex/age matched and were between 35 to 55 years old with a mean age of 51 and 45 years old for patients and healthy individuals respectively. All participants gave written consent prior to taking part in the study and the project was approved by the Ethics Committee of the Shiraz University of Medical Sciences.

*RNA Isolation and cDNA Synthesis*

Total RNA was prepared from peripheral blood after lysis with ammonium chloride and TRizol reagent (Invitrogen, USA). Prior to cDNA synthesis, the extracted RNAs were treated with DNase I (Invitrogen, USA) to avoid DNA contamination. cDNA was synthesized from 5μg of total RNA using the RevertAid First Strand cDNA Synthesis Kit (Fermentase, Lithuania).

*Real-Time PCR (RT-PCR)*

The expression of IL-23 and IL-27 gene transcripts was determined with real-time polymerase chain reaction (RT-PCR) using Syber Green PCR Master Mix (Applied Biosystems, USA). Each PCR reaction was carried out in a final volume of 25μl containing 12.5μl of 2xSYBR Green Master Mix, 0.2μl of each 10pmol/µl forward and reverse primers (designed with Primer Blast online software^22^ (as shown in table 1) and 8.1μl DEPC treated water. PCR amplification was performed in 40 cycles using the following sequence: 95ºC for 10 minutes, 95ºC for 15 seconds, 55ºC for 30 seconds and 60ºC for 60 seconds. Data were normalized based on beta-actin expression as housekeeping gene.

**Table 1:** Forward and reverse primers of β-actin, IL-23 and IL-27 genes for real-time PCR amplification

**Sequence**	**Primer**
AGCACTGTCTTGGCGTACAG	β-actin Forward
GGACTTCGAGCAAGAGATGG	β-actin Reverse
GTTCCCCATATCCAGTGTGG	IL-23 Forward
GGATCCTTTGCAAGCAGAAC	IL-23 Reverse
GAGCAGCTCCCTGATGTTTC	IL-27 Forward
AGCTGCATCCTCTCCATGTT	IL-27 Reverse

*Statistical Analysis*

The relative mRNA expressions of IL-23 and IL-27 in the peripheral blood of patients was evaluated using 2^-∆Ct^ method and compared with the corresponding values from the control samples using nonparametric Mann-Whitney U test/SPSS software version 15.5 (SPSS, USA). Analysis of gene expression with the pathological information of the patients was carried out using Kruskal-Wallis H tests. All graphs were presented using GraphPad Prism 5 (GraphPad Software, Inc. La Jolla, CA, USA).  Data were considered significant if P value was less than 0.05 with confidence interval of 95%..

## Results

*Histopathological* *Information of Patients*

Blood samples were taken a day before the surgery and then patient’s diseases were ascertained by pathological examination. All patients under investigation suffered  frominfiltrative ductal carcinoma of breast. Histopathological information is summarized in table 2.

**Table 2:** Histopathologic information of studied patients based on TNM staging which suggested by American Joint Committee on Cancer (AJCC)

**Factor **	**Frequency **	**Percentage**
Clinical Stage≠		
I	13	26
II	25	50
III	10	20
IV	2	4
Metastasis		
Without Metastasis	48	96
Metastatic	2	4
Tumor Grade		
I	11	22
II	23	46
III	13	26
IV	3	6
Tumor Size		
T1	16	32
T2	27	54
T3	5	10
T4	2	4
Tumor Necrosis		
Positive	35	70
Negative	15	30
Tumor Calcification		
Positive	40	80
Negative	10	20
Her2/neu		
Negative	13	26
1+	21	42
2++	7	14
3+++	9	18
Estrogen Receptor		
Negative	12	24
Positive	38	76
Progesterone Receptor		
Negative	19	38
Positive	31	62

TNM: T: Describes the size of the original (primary) Tumor, N: Describes nearby (regional) Lymph Node, M: describes distant metastasis.

*IL-23 and IL-27 mRNA Expressions in Peripheral Blood Mononuclear Cells (PBMCs)*

**Figure 1:**
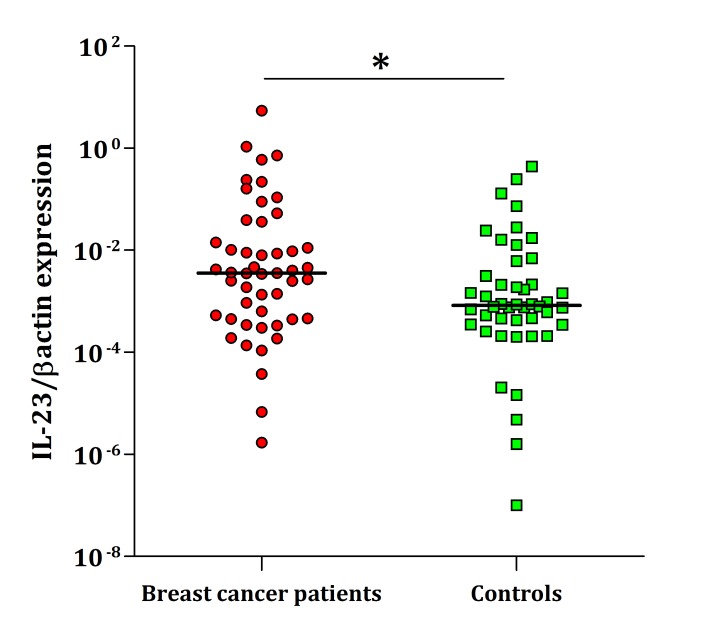
Real-time PCR analysis of IL-23 expression in PBMCs. As shown, the differences were statistically significant between breast cancer patients and normal control blood samples (P=0.032). The horizontal lines show the median of 2^-∆Ct^ of each group. The logarithmic scale 10 used for the x-axis.

**Figure 2:**
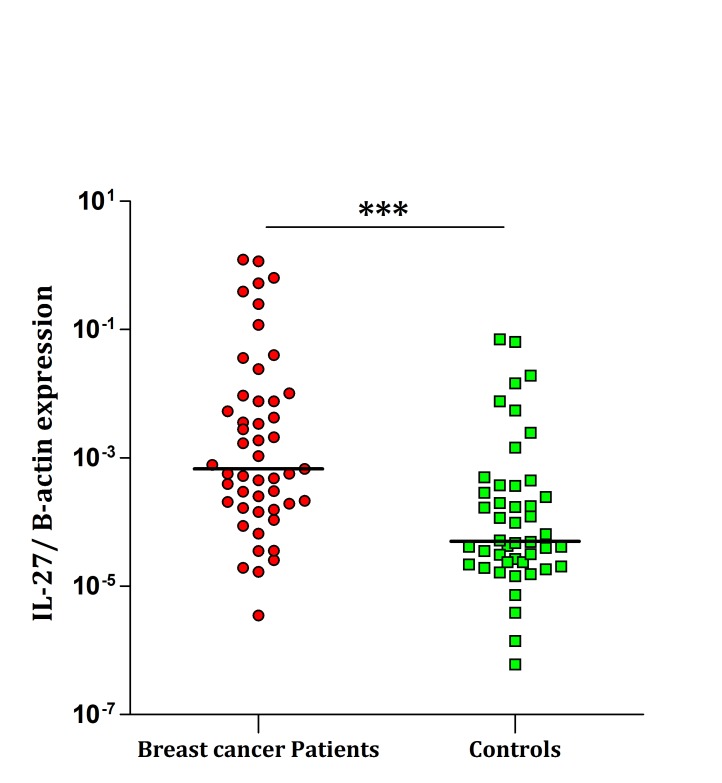
Real-time PCR analysis of IL-27 expression in PBMCs. The differences were statistically significant between breast cancer patients and normal control blood samples (P<0.0001). The horizontal lines show the median of 2^-∆Ct^ of each group. The logarithmic scale 10 used for the x-axis.

As depicted in figure 1, IL-23 shows significantly higher expression of mRNA in PBMCs of patients compared with the healthy individuals (P=0.032). Similarly, expression of IL-27 transcript in PBMCs were significantly higher in patients than healthy controls (P<0.0001) (figure 2). No correlation was found between the IL-23 and IL-27 transcripts and pathological stages, grades, ER, PR and HER-2 expression (data not presented).

*The Ratio of IL-23 to IL-27 in Peripheral Blood*

**Figure 3:**
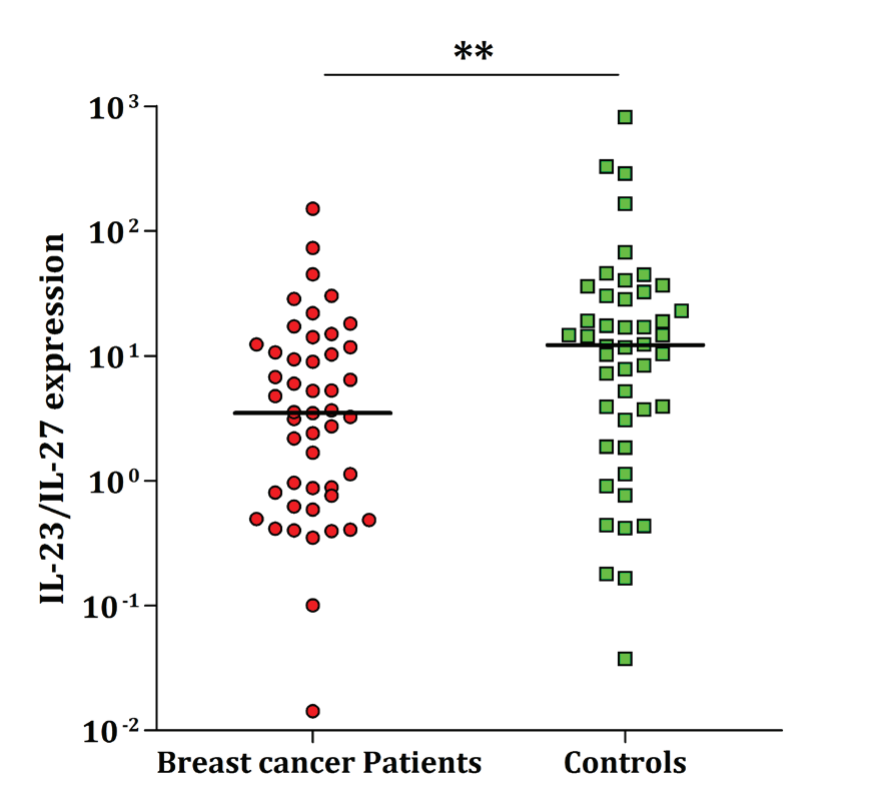
The expression ratio of IL-23 to IL-27 in breast cancer patients and normal individuals. It was 3.4-fold lower in patients compared to the controls (P=0.004). The horizontal lines show the median of ratio in each group. The logarithmic scale 10 used for the x-axis.    .

The ratio of IL-23 gene transcript to IL-27 in peripheral blood of healthy volunteers was 12.2 whereas it was 3.6 in patients; i.e. 3.4-fold lower in the studied patients compared with the normal individuals (P=0.004, figure 3).

## Discussion

Novel interleukin IL-12 related cytokines, IL-23 and IL-27, are reported to have potent antitumor activities. However, there are some controversial reports indicating the tumor promoting effects of IL-23.^2^ This study has established higher expression of IL-27 in peripheral blood of breast cancer patients compared with the healthy individuals. Such over expression of IL-27 in breast cancer patients might be an immune response to the tumor development.

Previous studies have shown that IL-27 inhibits the tumor growth of multiple myeloma cells via inhibition of angiogenesis.^23^ IL-27 activates STAT1 and STAT3 and enhances antitumor cellular and humoral immunity.^2^ Additionally, it has the ability of inhibiting both tumor growth and metastasis of murine melanoma cells.^24^ IL-27 antitumor functions mediated by antiangiogenic effects^24^ and activation of NK^25^ and CD8+ T cells.^26^ Accordingly, IL-27 has previously been introduced as a suitable candidate for antitumor immunotherapy.^27^ Recently, IL-27 is applied as a new antitumor cytokine with antiangiogenic and anti-inflammatory effects in clinical trials.^24^ However, it appears that there is a strong conflict between tumor cells and immune related cells during tumor development. As tumor cells exacerbate the pro-inflammatory and Th2 responses for their survival, the immune system reacts by up-regulating anti-inflammatory and Th1 responses such as IL-27 synthesis.

In contrast to IL-12 and IL-27, the role of IL-23 in cancer appears controversial.^28^ The exogenous and endogenous overexpression of IL-23 in the tumor microenvironment shows a converse function among previously published data.^2^ Several studies revealed that exogenous overexpression of IL-23 induced a potent anti-tumor response. Oniki et al. found that the antitumor effect of IL-23 was mediated by CD8+ T cells and IFN-gamma, whereas IL-27 mainly acts through natural killer cells in a mouse melanoma model.^25^ Lo et al. also documented that the antitumor mechanisms of IL-23 was mainly achieved by CD8^+^ T cells.^29^ Another study showed that the administration of IL-23 was successfully associated with significant suppression of fibrosarcoma growth with the therapeutic effects similar to those from IL-12 treatment. They also demonstrated that the potent antitumor activity of IL-23 was mainly mediated through Th1 responses which completely supported endogenous IL-12.^30^

Conversely, high level of the endogenous IL-23, which is related to human tumors, seems to promote inflammation and increasing angiogenesis.^31^ Langowski et al. found that the depletion of IL-23 leads to infiltration of cytotoxic T cells into the tumor tissues. They concluded that this cytokine is a critical molecular mediator between tumor-promoting pro-inflammatory responses and the failure of the adaptive immune response against tumors.^28^

In this investigation the expressions of IL-23 transcripts in PBMCs of breast cancer patients were studied. In the case of IL-23, data indicates higher expression of this cytokine in peripheral blood of breast cancer patients compare with the control group. Zhang et al. showed significant increase of mRNA expression of IL-17 and IL-23 in serum and also tumor tissues of patients with advanced gastric cancer.^32^ A significantly higher serum level of IL-23 in patients with colorectal cancer is strongly associated with overexpression of VEGF.^33^ However, Shime et al. reported increased expression of IL-23 mRNA in tumor microenvironment of patients with lung adenocarcinoma. They showed that lactic acid secreted by tumor cells enhances the transcription of IL-23 in the tumor-infiltrating immune cells.^34^ Similar result has been reported in human oral squamous cell carcinoma (HOSCC) cell lines and tissue.^35^

Although this study found that IL23 and IL27 transcripts significantly increased in peripheral blood of breast cancer patients, the IL-23/IL-27 ratio was significantly lower in PBMCs of breast cancer patients versus healthy volunteers which may lead to the defect in anti-tumor systemic immune responses. . 

## Conclusion

It is concluded that the overexpression of IL-23 and IL-27 gene transcripts may be an immune response to the tumor development. Data gathered in this study also shows that IL-23/IL-27 ratio may play an important role in cytokine-based immunotherapy against cancer. However, the final fate of a tumor depends on the complex relationship between tumor cells and cells of tumor microenvironment. Further research should be carried out to assess these cytokines in a larger sample size. 
